# Serum stromal cell-derived factor 1α as a prognostic indicator in elderly patients with acute myeloid leukemia receiving CAG-based chemotherapy

**DOI:** 10.3389/fonc.2024.1521179

**Published:** 2025-01-13

**Authors:** Zhenzhen Wang, Jing Yuan, Nan Zhou, Jianfeng Zhang

**Affiliations:** ^1^ Department of Hematology, the Second Hospital of Hebei Medical University, Hebei, Shijiazhuang, China; ^2^ The Second Department of General Surgery, the Fourth Hospital of Hebei Medical University, Hebei, Shijiazhuang, China

**Keywords:** stromal-cell-derived factor 1 alpha (SDF-1α), acute myeloid leukemia (AML), elderly, CXC chemokine receptor type 4 (CXCR4), chemotherapy response

## Abstract

**Background:**

Stromal-cell-derived factor 1 (SDF-1) plays a crucial role in hematopoiesis and has been implicated in acute myeloid leukemia (AML) pathogenesis. Understanding its relationship with chemotherapy outcomes could lead to improved therapeutic approaches for elderly AML patients.

**Methods:**

This study retrospectively analyzed the medical records of elderly AML patients (n = 187) and compared serum SDF-1α levels with age-matched controls (n = 120). Patients received CAG (cytarabine, aclarubicin, and G‐CSF)-based chemotherapy, and serum SDF-1α levels were assessed using ELISA.

**Results:**

Serum SDF-1α levels were significantly elevated in elderly AML patients compared to controls (p < 0.001). Receiver operating characteristic (ROC) analysis confirmed its diagnostic relevance, revealing the area under the ROC curve (AUC) of 0.76. Factors such as age, French-American-British (FAB) classification, Eastern Cooperative Oncology Group (ECOG) performance status, primary AML status, white blood cell count, and bone marrow blast cell ratio, were confirmed to be prognostically relevant. Serum SDF-1α levels were elevated in patients who did not achieve complete remission (NCR) compared to those in complete remission (CR). ROC analysis further highlighted the predictive capability of serum SDF-1α for chemotherapy responsiveness. Independent predictors of treatment failure included age, FAB classification, ECOG status, and serum SDF-1α levels. Following chemotherapy, serum SDF-1α levels decreased in patients in CR but remained unchanged in those in NCR. Higher baseline levels of SDF-1α were associated with shorter overall survival.

**Conclusions:**

Elevated serum SDF-1α levels in elderly AML patients are associated with poor chemotherapy response and shorter survival. Baseline serum SDF-1α levels could serve as a prognostic marker for CAG-based treatment outcomes.

## Introduction

Acute myeloid leukemia (AML) is a prevalent malignancy in the elderly, typically diagnosed at around 70 years (1, 2). Despite advances in treatment, survival rates for older adults have not improved significantly, with less than 10% of patients over 65 surviving past five years post-diagnosis (1). The poor prognosis in older AML patients is multifaceted, stemming from age-related factors such as reduced performance status, comorbidities, and increased likelihood of severe treatment complications (3). Additionally, the elderly have a distinct biological and cytogenetic landscape, often presenting with unfavorable genetic mutations, secondary AML, or treatment-related AML, further complicating their therapeutic response (4). These complexities necessitate a deeper investigation into the factors affecting treatment tolerance and the development of more individualized treatment approaches for the elderly with AML.

Stromal-cell-derived factor 1 (SDF-1, CXCL12) is a CXC-chemokine continually produced and affects various physiological pathways, such as the development of the embryo and the homeostasis of organs (5–7). The pleiotropic chemokine is generated/secreted by a variety of organs, such as the lung, liver, bone marrow, and skin. SDF-1 stimulates G protein-coupled receptors, CXC chemokine receptor type 7 (CXCR7) and CXCR4, which have roles in inflammation, neurogenesis, hematopoiesis, angiogenesis, cancer metastasis, and HIV infection. SDF-1α plays a key role in various biological processes, including immune response, cell differentiation, tissue repair, and tumorigenesis (8–10) It facilitates the migration and differentiation of immune cells and has been implicated the migration, homing, and mobilization of hematopoietic cells, playing a vital role in the maintenance and regulation of the bone marrow niche (11). SDF-1 also contributes to angiogenesis, as seen in reduced vascularization in osteosarcoma upon SDF-1 suppression (12). Interestingly, serum SDF-1α levels have been found to exhibit a significant positive correlation with increasing age (13). A previous study demonstrated that patients with AML exhibited increased total SDF-1 concentrations in their peripheral blood, while simultaneously showing decreased levels of functionally active SDF-1 (14). While serum SDF-1 is not exclusive to any single AML subtype, certain studies have observed higher levels in specific subtypes. For instance, AML M4 and M5 (acute myelomonocytic leukemia and acute monocytic leukemia) tend to show elevated SDF-1 levels, potentially due to their higher percentage of monocytic cells, which are known to express high levels of CXCR4 and are particularly responsive to SDF-1 signaling (15).

CXCR4 and its ligand, SDF-1, constitute a critical axis in AML, involved in the navigation and maintenance of leukemia cells within the bone marrow niche, a sanctuary that offers protection and sustenance to both malignant and healthy hematopoietic stem cells. The prognostic significance of CXCR4 expression on leukemia cells in AML patients has been recognized, as varying levels of expression correlate with disease outcomes (16, 17). Targeting CXCR4 with antagonists to dislodge leukemia cells from the BM has shown promise, making these cells more vulnerable to treatment. This strategy has paved the way for the use of CXCR4 antagonists in clinical trials, where they have been combined with conventional chemotherapy and other agents to potentially improve therapeutic efficacy.

Our study aims to investigate the concentration of SDF-1α in the serum of elderly AML patients compared to that in healthy individuals, analyze its correlation with chemotherapy outcomes, and observe the fluctuations in serum SDF-1α levels before and after chemotherapy. Understanding these relationships could pave the way for more effective and individualized treatment regimens for elderly AML patients.

## Methods

### Study design and patient selection

This study retrospectively analyzed the medical records and follow-up data of elderly patients diagnosed with AML at the Second Hospital of Hebei Medical University. Inclusion criteria were based on the diagnostic standards outlined in the Chinese Society of Hematology’s Guidelines for the Diagnosis and Treatment of Adult Acute Myeloid Leukemia (2011 edition). Patients aged 60 years or older, without prior treatment, including radiation, chemotherapy, targeted therapy, or hormonal therapy, were included. Exclusion criteria comprised patients with a history of AML retreatment, concurrent malignancies, severe organ dysfunction, acute cardiovascular or cerebrovascular events, incomplete clinical data, or those who expired during chemotherapy. The study was approved by the Second Hospital of Hebei Medical University.

The study comprised two primary groups, 120 elderly controls and 187 elderly AML patients who had received chemotherapy (**Supplementary Figure S1**). Serum levels of SDF-1α were measured in both groups. AML patients were further categorized into two subgroups based on treatment response, complete remission (CR, n=79) and non-complete remission (NCR, n=108). For both CR and NCR groups, serum SDF-1α levels were measured at baseline and post-chemotherapy. The serum SDF-1α levels were utilized for subsequent analyses. Multivariate logistic analysis and overall survival analysis were conducted.

### Clinical and laboratory assessments

Patients were classified according to the French-American-British (FAB) classification system and assessed using the Eastern Cooperative Oncology Group (ECOG) performance status scale. Serum SDF-1α levels were assessed by an enzyme-linked immunosorbent assay kit (R&D Systems, USA).

### Chemotherapy regimen

The chemotherapy regimen employed was based on CAG, comprising the following specific treatments: idarubicin 14 mg/m^2^ administered intravenously once daily on days 1-4, cytarabine 10 mg/m^2^ administered subcutaneously once every 12 hours on days 1-14, and granulocyte colony-stimulating factor 200 μg/m^2^ administered subcutaneously on days 1-14.

### Response evaluation

Complete remission (CR) was defined as per the criteria outlined in the standard diagnostic and therapeutic criteria for hematologic diseases. CR was characterized by the absence of clinical symptoms and signs attributable to leukemia cell infiltration, with hemoglobin levels ≥100 g/L (in males) or ≥90 g/L (in females and children), absolute neutrophil counts ≥1.5×10^9^/L, platelet counts ≥100×10^9/L, absence of leukemia cells in peripheral blood smears, and bone marrow blast cell ratio ≤5%. Overall survival (OS) was calculated from the end of chemotherapy to the date of death, loss to follow-up, or the end of the study period.

### Statistical analysis

**Figure 1:**
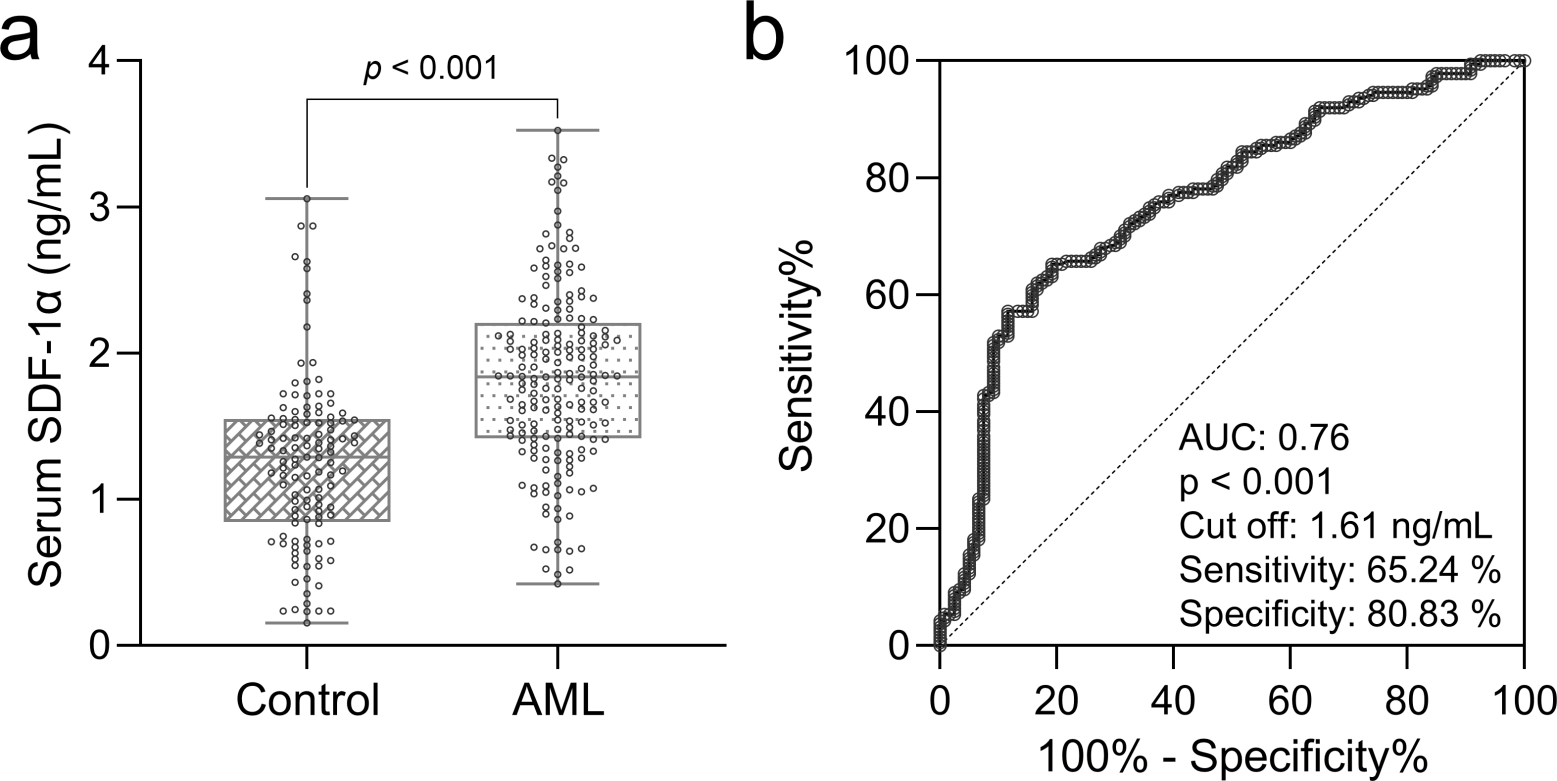
Elevated serum SDF-1α in elderly with AML. **(A)** Box plots comparing serum SDF-1α levels between elderly patients with acute myeloid leukemia (AML) (n = 187) and healthy controls (n = 120), with p-values derived from the Mann-Whitney test. **(B)** Features ROC analysis assessing the ability of serum SDF-1α to differentiate elderly individuals with AML from controls.

**Figure 2:**
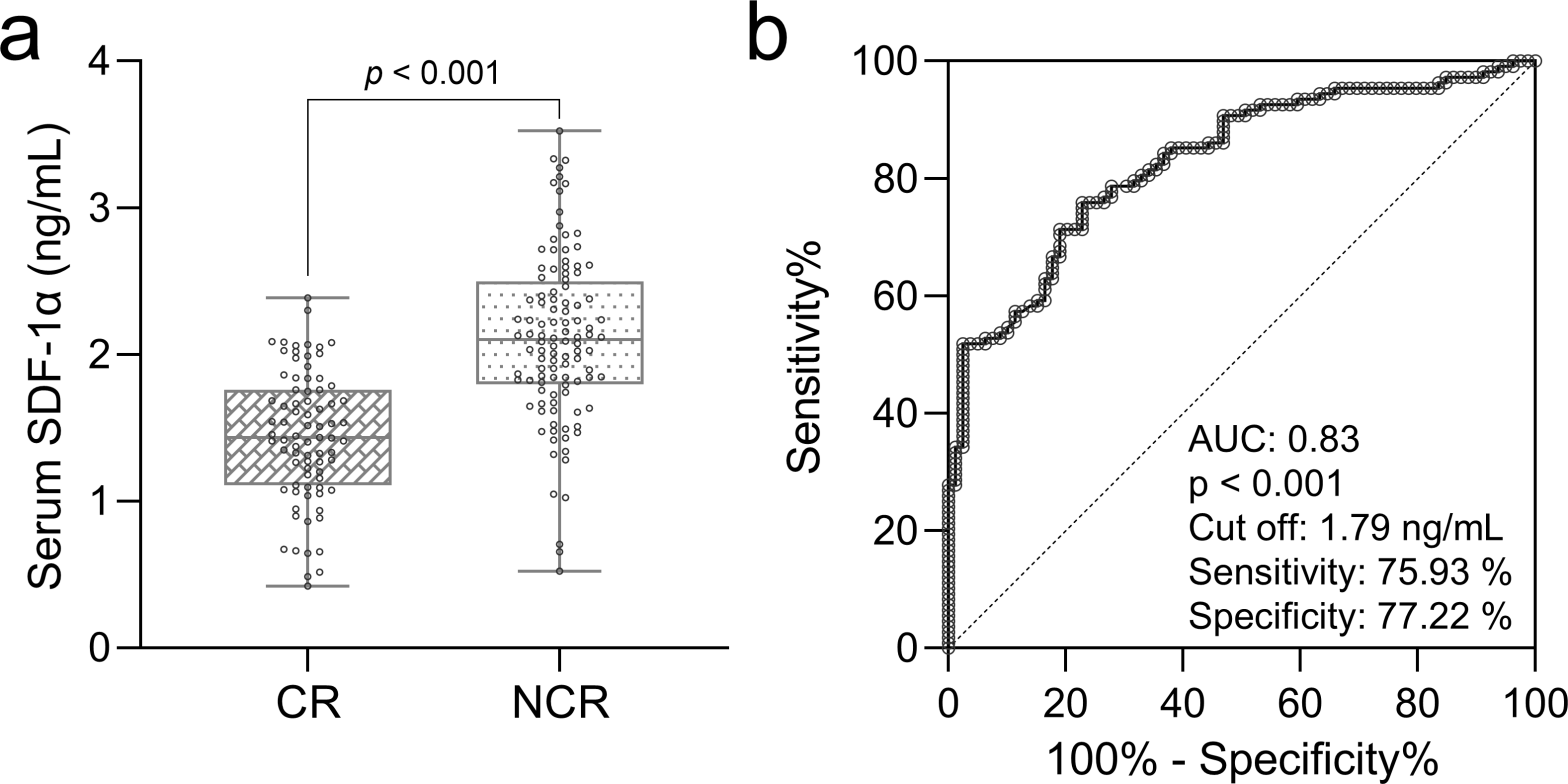
Prognostic role of serum SDF-1α in AML chemosensitivity. **(A)** Box plots show baseline serum SDF-1α levels compared between elderly AML patients achieving complete remission (CR, n = 79) and those not achieving remission (NCR, n = 108), with significance tested by the Mann-Whitney test. **(B)** ROC analysis predicts complete remission after chemotherapy based on baseline serum SDF-1α levels in these patients.

Data were presented as median ± standard deviation (SD). Student’s t-test and chi-square test were utilized for continuous and categorical variables, respectively. Regarding the odds ratio (OR) estimation, we employed multivariate logistic regression analysis to identify risk factors affecting short-term prognosis in elderly AML patients after chemotherapy. The dependent variable was complete remission status (CR = 0, NCR = 1), while the independent variables were the factors showing significant differences in **Table 1**. The formula for odds ratio is OR = (a/c)/(b/d), where ‘a’ is the number of exposed cases, ‘b’ is the number of exposed controls, ‘c’ is the number of unexposed cases, and ‘d’ is the number of unexposed controls. For the Receiver operating characteristic (ROC) analysis, we utilized standard methods available in our statistical software. In **Figure 1**, AML was set as the disease group and Control as the reference group. Similarly, for **Figure 2**, NCR was designated as the disease group and CR as the reference group. The software then automatically generated the ROC curves and calculated the Area Under the Curve (AUC). Regarding the choice of statistical tests, we used Fisher’s exact test for comparisons between two groups with binary variables. For comparisons involving three or more categories, we employed the Chi-square test. We will clearly indicate in **Table 1** which test was used for each parameter to improve clarity. Our analysis identified several independent risk factors for failure to achieve complete remission in elderly AML patients after chemotherapy. These include age more than 75 years old, FAB subtypes M4/M5/M6, ECOG status 3-4, and serum SDF-1α levels more than 1.79 ng/mL. Statistical analyses were conducted using SPSS version 20 (IBM Corp., USA), with p-values <0.05 considered statistically significant.

**Table 1:** Analysis of single factors affecting the chemosensitivity in elderly patients with acute myeloid leukemia (AML).

Factors	CR (n = 79)	NCR (n = 108)	p value
Gender			
Male	43 (54.4%)	61 (56.5%)	0.882
Female	36 (45.6%)	47 (43.5%)	
Age (years)			
60-69	30 (37.9%)	18 (16.7%)	< 0.001
70-79	34 (43.1%)	47 (43.5%)	
≥ 80	15 (19.0%)	43 (39.8%)	
FAB classification			
M0	4 (5.1%)	0 (0%)	< 0.001
M1	5 (6.3%)	10 (9.3%)	
M2	26 (32.9%)	13 (12.0%)	
M3	21 (26.5%)	27 (25.0%)	
M4	19 (24.1%)	42 (38.9%)	
M5	4 (5.1%)	15 (13.9%)	
M6	0 (0%)	1 (0.9%)	
ECOG (Eastern Cooperative Oncology Group)			
1	25 (31.6%)	18 (16.7%)	0.002
2	39 (49.4%)	43 (39.8%)	
3	15 (19.0%)	44 (40.7%)	
4	0 (0%)	3 (2.8%)	
Primary AML			
Yes	77 (97.5%)	95 (87.9%)	0.027
No	2 (2.5%)	13 (12.1%)	
White blood cell count (* 10^9^/L)			
< 50	68 (86.1%)	74 (68.5%)	0.006
≥ 50	11 (13.9%)	34 (31.5%)	
Blood platelet count (* 10^9^/L)			
< 20	35 (44.3%)	58 (53.7%)	0.237
≥ 20	44 (55.7%)	50 (46.3%)	
Bone marrow blast cell ratio (%)			
< 50	60 (75.9%)	36 (33.3%)	< 0.001
≥ 50	19 (24.1%)	72 (66.7%)	

The data were shown as n (percentage). The comparisons of data between the two groups were done by Fisher’s exact test or Chi-square test.

CR, complete remission; NCR, without complete remission.

## Results

### Elevated serum SDF-1α in elderly with AML

In a retrospective analysis, serum SDF-1α levels were significantly higher in elderly patients with AML (n = 187) compared to age-matched control subjects (n = 120, p< 0.001, **Figure 1A**). The diagnostic utility of serum SDF-1α was further substantiated through ROC curve analysis, which yielded an AUC of 0.76, with an optimal cut-off value at 161 ng/mL, sensitivity of 65.24%, and specificity of 80.83% (**Figure 1B**), suggesting that SDF-1α could be a valuable biomarker for distinguishing AML in the elderly population.

### Factors influencing chemosensitivity in elderly AML patients

Further examination of the single-factor analysis identified critical factors impacting chemosensitivity following two cycles of induction chemotherapy. Of the patients, 79 achieved complete remission (CR), while 108 did not respond completely (NCR) or died. We found statistically significant differences in short-term prognosis between the two groups based on age, FAB classification, Eastern Cooperative Oncology Group (ECOG) performance status, primary versus secondary AML, white blood cell count, and bone marrow blast cell ratio (**Table 1**). Gender and blood platelet count did not show a significant impact on chemosensitivity. Age, with a p-value of < 0.001, FAB classification, with specific subtypes such as M0 showing a significant difference, ECOG performance status (p = 0.002), primary AML status (p = 0.027), white blood cell count (p = 0.006), and bone marrow blast cell ratio (p < 0.001), were found to be prognostically relevant, indicating their potential as predictors for treatment response in this patient population.

### Prognostic role of serum SDF-1α in AML chemosensitivity

To analyze the prognostic value of serum SDF-1α levels in chemosensitivity of elderly patients with AML, we divided patients into those who achieved complete remission (CR, n = 79) and those who did not (NCR, n = 108), based on their response to two cycles of induction chemotherapy. Baseline serum levels of SDF-1α were compared between the two groups, revealing significantly higher concentrations in patients who did not achieve complete remission (p < 0.001, **Figure 2A**). A ROC analysis was conducted using baseline serum SDF-1α levels to predict chemosensitivity (short-term prognosis) in these patients (**Figure 2B**). The analysis indicated a strong prognostic value with an AUC of 0.83, suggesting that baseline serum SDF-1α is a potential predictor of treatment response in elderly AML patients.

### Independent risk factors for chemotherapy response in elderly AML

In the multivariate logistic regression analysis, factors influencing the likelihood of achieving complete remission after two cycles of induction chemotherapy were identified (**Table 2**). The analysis, which considered CR and NCR as dependent variables, revealed several independent risk factors for the absence of complete remission. Patients aged over 75 years had an OR of 2.364, indicating more than double the risk of not achieving CR compared to younger patients. Notably, those with FAB classification M4/M5/M6 had a markedly higher risk (OR of 4.217), and ECOG performance status of 3-4 was associated with an OR of 3.892. Additionally, a serum SDF-1α level higher than 1.79 ng/mL was associated with a 46% increase in the risk of not achieving CR (OR of 1.462). The presence of secondary AML, a white blood cell counts greater than 50 × 10^9^/L, and a bone marrow blast cell ratio over 50% also appeared to influence chemosensitivity, but not to a statistically significant extent. These findings suggest that age, FAB classification, ECOG status, and serum SDF-1α levels are significant predictors of chemotherapeutic response in the elderly AML population.

**Table 2:** Multivariate logistic analysis for the chemosensitivity in elderly patients with acute myeloid leukemia (AML).

	OR	95% CI	p value
Age more than 75 years old	2.364	1.526 to 6.114	0.009
FAB with M4/M5/M6	4.217	3.085 to 9.893	0.002
ECOG with 3-4	3.892	2.174 to 8.602	0.006
Secondary AML	1.867	0.914 to 3.225	0.125
White blood cell count more than 50 * 10^9^/L	2.073	0.946 to 3.148	0.087
Bone marrow blast cell ratio more than 50%	1.684	0.853 to 2.795	0.119
Seum SDF-1α more than 1.79 ng/mL	1.462	1.098 to 1.883	0.027

OR, Odds Ratio; CI, confidence interval.

ECOG, Eastern Cooperative Oncology Group.

### Serum SDF-1α response to chemotherapy in elderly AML patients

**Figure 3:**
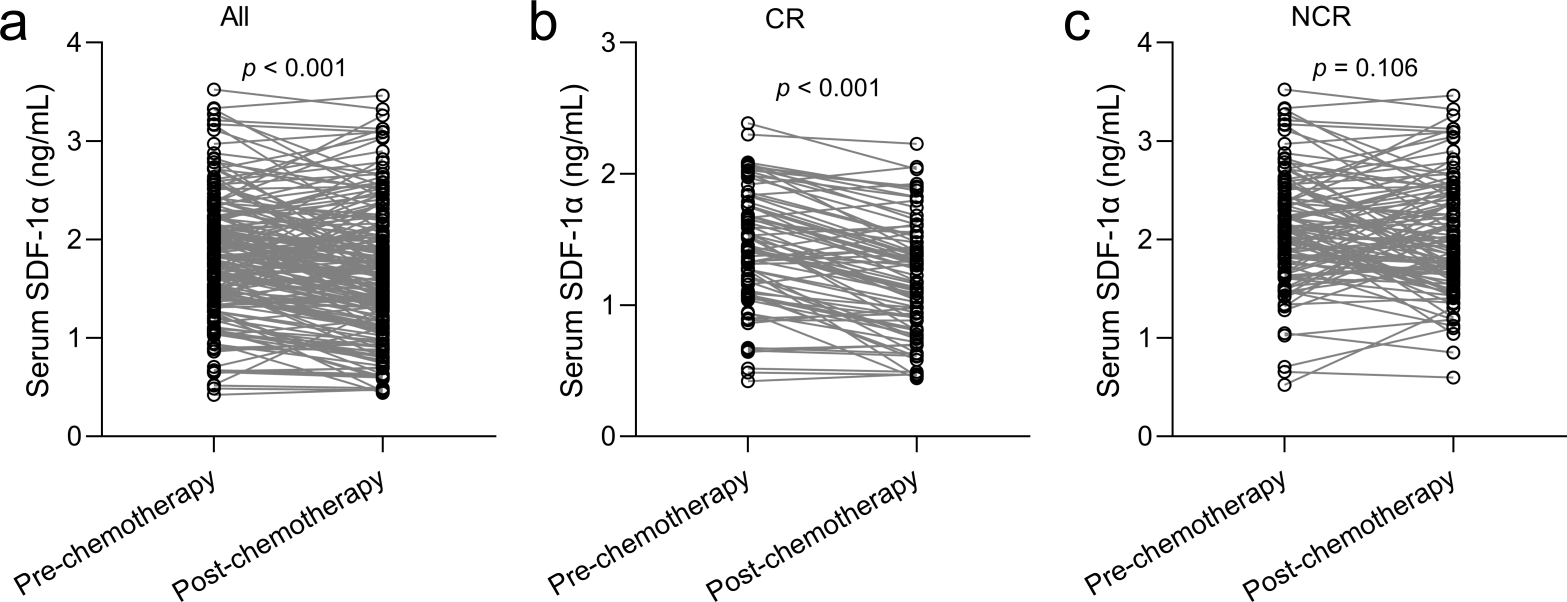
Serum SDF-1α response to chemotherapy in elderly AML patients. **(A)** Changes in serum SDF-1α post-chemotherapy are tracked for elderly AML patients, with CR **(B)** and without CR **(C)**, analyzed using paired t-tests for statistical significance.

We next investigated the variations in serum SDF-1α levels among AML patients before and after chemotherapy. A decrease in serum SDF-1α was observed post-treatment across both all patients (**Figure 3A**, p < 0.001) and patients who achieved CR (**Figure 3B**, p < 0.001). Conversely, in patients who did not reach CR (NCR, **Figure 3C**), serum SDF-1α levels did not change significantly post-chemotherapy (p = 0.105). These findings suggest a correlation between serum SDF-1α levels and the sensitivity of elderly AML patients to chemotherapy, where a decrease in SDF-1α is associated with a positive response to treatment.

### Baseline serum SDF-1α and long-term survival in elderly AML patients

**Figure 4:**
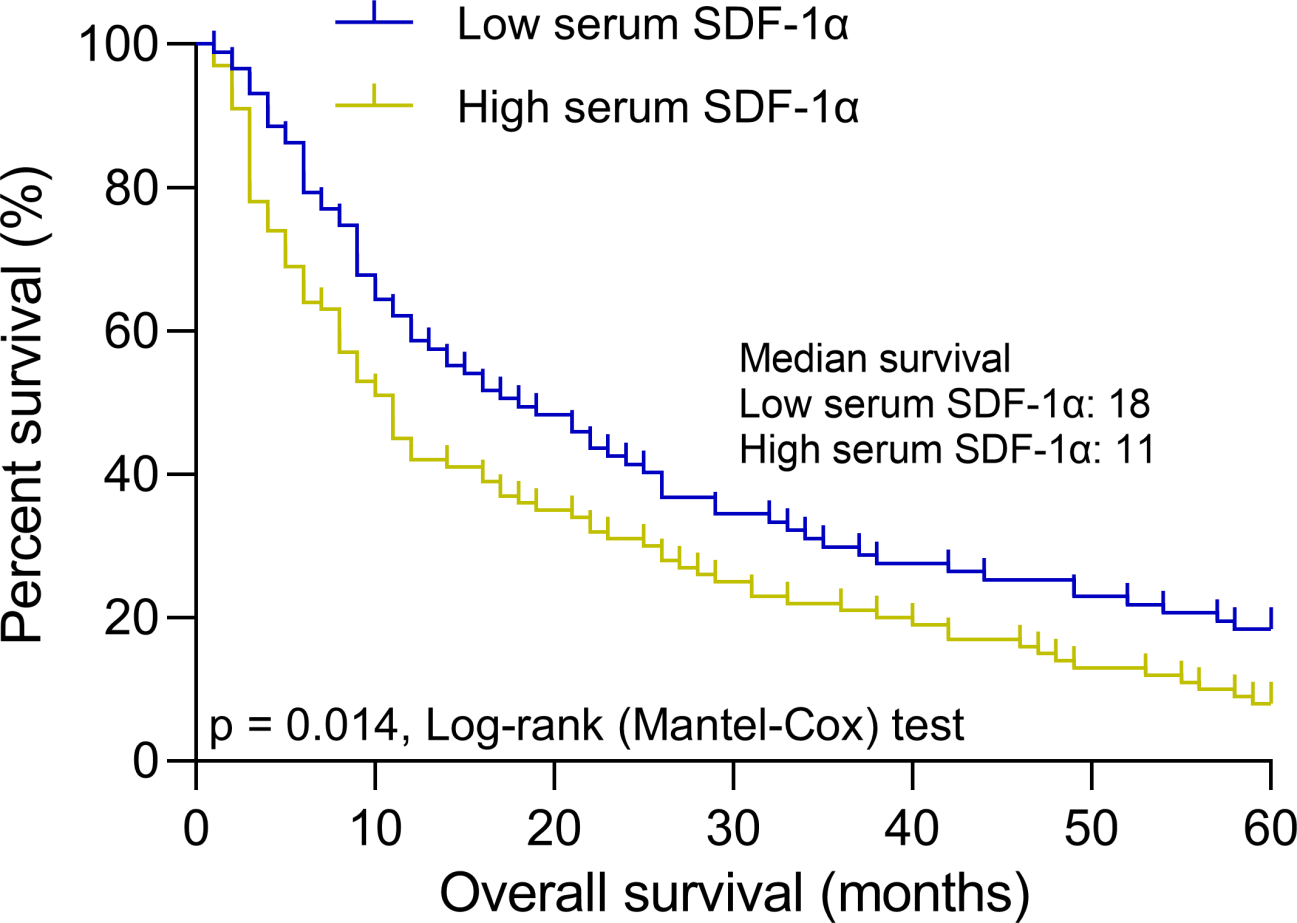
Baseline serum SDF-1α and long-term survival in elderly AML patients. Patients were stratified into low (n = 87) and high (n = 100) serum SDF-1α groups based on a cut-off of 1.79 ng/mL for predicting complete remission after chemotherapy. The panel illustrates 5-year overall survival curves for these groups, highlighting the impact of baseline serum SDF-1α levels on long-term outcomes.

To confirm the prognostic significance of baseline serum SDF-1α levels in elderly AML patients, we utilized a cut-off value of 1.79 ng/mL to predict complete remission post-chemotherapy. The cohort was stratified into a low serum SDF-1α group (n = 87) and a high serum SDF-1α group (n = 100). Over a 5-year follow-up period, the OS curves clearly demonstrated a significant difference between the two groups. Patients in the low SDF-1α group had a median overall survival of 18 months, whereas those in the high SDF-1α group had a notably shorter median OS of only 11 months (**Figure 4**). This disparity suggests a strong association between baseline serum SDF-1α levels at diagnosis and long-term outcomes in elderly AML patients, with lower levels indicating a more favorable prognosis.

## Discussion

The discovery of effective biomarkers for elderly AML patients holds immense clinical importance due to the unique challenges this group faces. AML in older adults is characterized by a lower survival rate, with less than 10% living beyond five years post-diagnosis, exacerbated by age-related declines in physical health, comorbidities, and more frequent adverse reactions to aggressive treatments. Additionally, the biological and genetic profiles of AML in the elderly often include unfavorable mutations and complexities such as secondary or treatment-related AML, which complicate response to standard therapies. In this study, we found that the serum SDF-1α, with its significant roles in immune cell migration, tissue repair, and tumor microenvironment regulation, could serve as a critical indicator of treatment response and overall prognosis. Elevated SDF-1α levels were identified as significant both diagnostically and prognostically. Serum SDF-1α not only differentiated AML patients from controls with notable accuracy (AUC of 0.76), but also emerged as a predictive marker for chemotherapy outcomes, with higher levels correlating with NCR and shorter overall survival. Several factors, including age, FAB classification, ECOG performance status, and initial serum SDF-1α levels, were statistically significant in predicting chemosensitivity and risk of treatment failure. We therefore propose a simple illustrative model, which begins with patient assessment, considering factors such as age (with >75 years as a risk factor), FAB classification (M4/M5/M6 as risk factors), ECOG performance status (3-4 as risk factors), and serum SDF-1α level. Risk stratification follows, with serum SDF-1α > 1.79 ng/mL indicating high risk and ≤ 1.79 ng/mL suggesting lower risk. Importantly, a post-chemotherapy decrease in serum SDF-1α levels was associated with positive response to treatment, emphasizing its potential as a biomarker for monitoring treatment efficacy and predicting long-term survival in this vulnerable population.

SDF-1 activates Akt and ERK pathways, promoting invasion, metastasis, cell cycle progression, epithelial-mesenchymal transition, proliferation, and migration in various cancer types, including lung cancer, AML and glioblastoma (18, 19). Additionally, in breast cancer, elevated SDF-1 levels activate the NF-κB pathway, promoting epithelial-mesenchymal transition and the emergence of cancer stem cell-like characteristics (20). In AML, SDF-1 plays a crucial role through its interaction with the CXCR4 receptor, influencing several aspects of the disease. Primarily, SDF-1α/CXCR4 interaction facilitates the homing and retention of leukemic stem cells within the protective bone marrow microenvironment, promoting their survival, proliferation, and contributing to chemotherapy resistance (21, 22). This interaction triggers key signaling pathways such as PI3K/Akt and MAPK/ERK that enhance cell survival and drug resistance, posing significant challenges in treatment and contributing to high relapse rates (23–26). Our results underscore the diagnostic potential of serum SDF-1α, as evidenced by a significant elevation in patients with AML compared to healthy controls. The ROC curve analysis yielded an AUC of 0.76, demonstrating a reasonably good diagnostic performance. This is particularly valuable given the complexity of diagnosing AML in the elderly, who often present with ambiguous clinical manifestations and have comorbid conditions that may confound standard diagnostic criteria. Our findings support earlier studies suggesting that in AML, elevated levels of CXCR4-expressing microparticles and SDF-1, which correlate with higher white blood cell counts, play a significant role in disease progression and may serve as potential diagnostic markers (14). In addition, SDF-1 gene polymorphisms and CXCR4 expression in AML patients were found to correlate with higher disease risk and poorer prognosis (15), suggesting their potential as targets for therapy and markers in diagnostic evaluations. However, the sensitivity and specificity achieved in this study suggest that while SDF-1α is promising, it is not definitive on its own. It would be prudent to use SDF-1α in conjunction with other diagnostic tools to improve overall accuracy, thereby facilitating early and accurate diagnosis, which is crucial for the effective management of AML.

Further insights were gained from examining factors that influence chemosensitivity in elderly AML patients. The single-factor analysis highlighted several parameters including age, FAB classification, ECOG performance status, and primary versus secondary AML status as significant. A comprehensive analysis highlighted the complexity of treating older AML patients, showing that while age alone should not dictate the suitability for intensive therapy, it remains an important factor in evaluating treatment options and outcomes, with various studies indicating differing impacts of therapy types on survival depending on age and clinical characteristics (27). In a study of 1252 AML patients, they reported a significant correlation between performance status (PS) and various clinical outcomes (28). Notably, as PS increased, median age also rose, and there was an associated increase in healthcare utilization, comorbidity scores, and worsened laboratory values (higher white blood cell counts and creatinine, lower albumin). These findings underline the importance of considering performance status as a significant factor in the clinical management and treatment strategy decisions for AML patients. Analysis of 1690 AML patients undergoing allogeneic stem cell transplantation revealed that the FAB classification, particularly types M6/M7, is predictive of worse outcomes, including decreased leukemia-free survival and increased nonrelapse mortality, highlighting its continued prognostic relevance in AML, NOS patients (29). The prognostic variability by FAB classification and ECOG status underscores the heterogeneous nature of AML and the need for individualized treatment approaches.

Based on the important roles of SDF-1α/CXCR4 signaling in AML chemotherapy resistance, a previous study showed that inhibition of CXCR4 disrupted the SDF-1α/CXCR4 signaling pathway, which plays a crucial role in protecting AML cells from chemotherapy-induced apoptosis by mediating their interaction with the bone marrow microenvironment (21). By antagonizing this pathway, it could enhance the sensitivity of AML cells to chemotherapy, potentially improving treatment outcomes. In evaluating the prognostic role of serum SDF-1α in relation to chemosensitivity, our study provides novel insights. Higher baseline SDF-1α levels were notably associated with a lower likelihood of achieving complete remission post-chemotherapy. This finding suggests a potential mechanistic role of SDF-1α in mediating resistance to chemotherapeutic agents, possibly through the promotion of survival pathways in leukemic cells. The strong prognostic value indicated by an AUC of 0.83 for predicting chemosensitivity further reinforces the potential of SDF-1α as a predictive biomarker, suggesting that its baseline measurement could inform treatment planning and prognosis. The multivariate logistic regression analysis identified several independent risk factors that affect the likelihood of achieving complete remission. Notably, older age, advanced FAB classifications, and poorer ECOG performance status significantly increased the risk of poorer chemotherapy outcomes. Moreover, a higher serum SDF-1α level was associated with an increased risk of not achieving complete remission, highlighting its role as a critical biomarker in clinical prognostication. These factors could be utilized to construct a prognostic model that predicts treatment outcomes, enabling clinicians to tailor aggressive or conservative treatment strategies based on a patient individual risk profile.

Previous prognostic studies demonstrated that SDF-1 expression was significantly correlated with poor prognosis of breast cancers (30). High pre-treatment levels and increasing post-treatment levels of SDF-1α are independent prognostic factors associated with poor progression-free and overall survival in esophageal squamous cell carcinoma patients undergoing concurrent chemoradiotherapy (31). In addition, elevated CXCR4 expression on leukemia cells were also found to be associated with a poor prognosis in patients with AML (32). By analyzing the dynamic response of serum SDF-1α levels to chemotherapy, we found a decrease in SDF-1α post-treatment was associated with a positive response to chemotherapy, evident from the significant reductions in patients who achieved CR. This contrasts with those who did not achieve remission, where SDF-1α levels remained largely unchanged. These observations suggest that monitoring SDF-1α levels during treatment could serve as an indicator of therapeutic effectiveness, providing real-time feedback on patient response to chemotherapy. Lastly, our longitudinal analysis of baseline serum SDF-1α and its impact on long-term survival outcomes provided compelling evidence of its prognostic significance. Patients with lower baseline levels of SDF-1α exhibited significantly longer median overall survival, suggesting that lower SDF-1α levels at diagnosis are indicative of a more favorable prognosis.

However, for further studies, we may combine with other diagnostic strategies to enhance the predictive and prognostic value of SDF-1. Additionally, targeting the SDF1/CXCR4 axis represents an exciting avenue for therapeutic intervention. Several CXCR4 inhibitors, such as plerixafor (AMD3100) and BL-8040, have shown promise in preclinical and early clinical studies for AML. However, challenges remain, particularly in balancing the inhibition of leukemic cell homing while preserving normal hematopoietic stem cell function. The critical role of the SDF1/CXCR4 axis in normal stem cell trafficking and tissue regeneration necessitates careful consideration of potential side effects. While our study focused on elderly AML patients, the prognostic significance of SDF1/CXCR4 in pediatric AML may differ due to the distinct biological characteristics of childhood leukemia. Studies in younger cohorts have suggested that high CXCR4 expression correlates with poor prognosis, but the relationship might not be as straightforward as in elderly patients. Our findings could potentially be extrapolated to other hematological malignancies and solid tumors where the SDF1/CXCR4 axis plays a role in disease progression and metastasis, such as lymphomas, multiple myeloma, and breast cancer. However, tumor-specific studies would be necessary to validate the prognostic value of SDF1 in these contexts.

## Conclusions

In conclusion, the multifaceted roles of SDF-1α highlighted in our study emphasize its potential as a biomarker for diagnosing, predicting treatment response, and prognosticating in elderly AML patients. These findings warrant further exploration into the mechanisms by which SDF-1α contributes to AML pathophysiology and treatment outcomes. Moreover, prospective clinical trials are needed to validate the clinical utility of incorporating SDF-1α into routine practice, which could revolutionize the management approach to AML in the elderly, ultimately improving both therapeutic outcomes and quality of life for this vulnerable patient population.

## Data Availability

The original contributions presented in the study are included in the article/**Supplementary Material**. Further inquiries can be directed to the corresponding author.
