# Supplementary figures and images for: Serum stromal cell-derived factor 1α as a prognostic indicator in elderly patients with acute myeloid leukemia receiving CAG-based chemotherapy
Source: Front Oncol. 2025 Jan 13;14:1521179. doi: 10.3389/fonc.2024.1521179 (PMC11769979; doi:10.3389/fonc.2024.1521179)

**Supplementary materials**


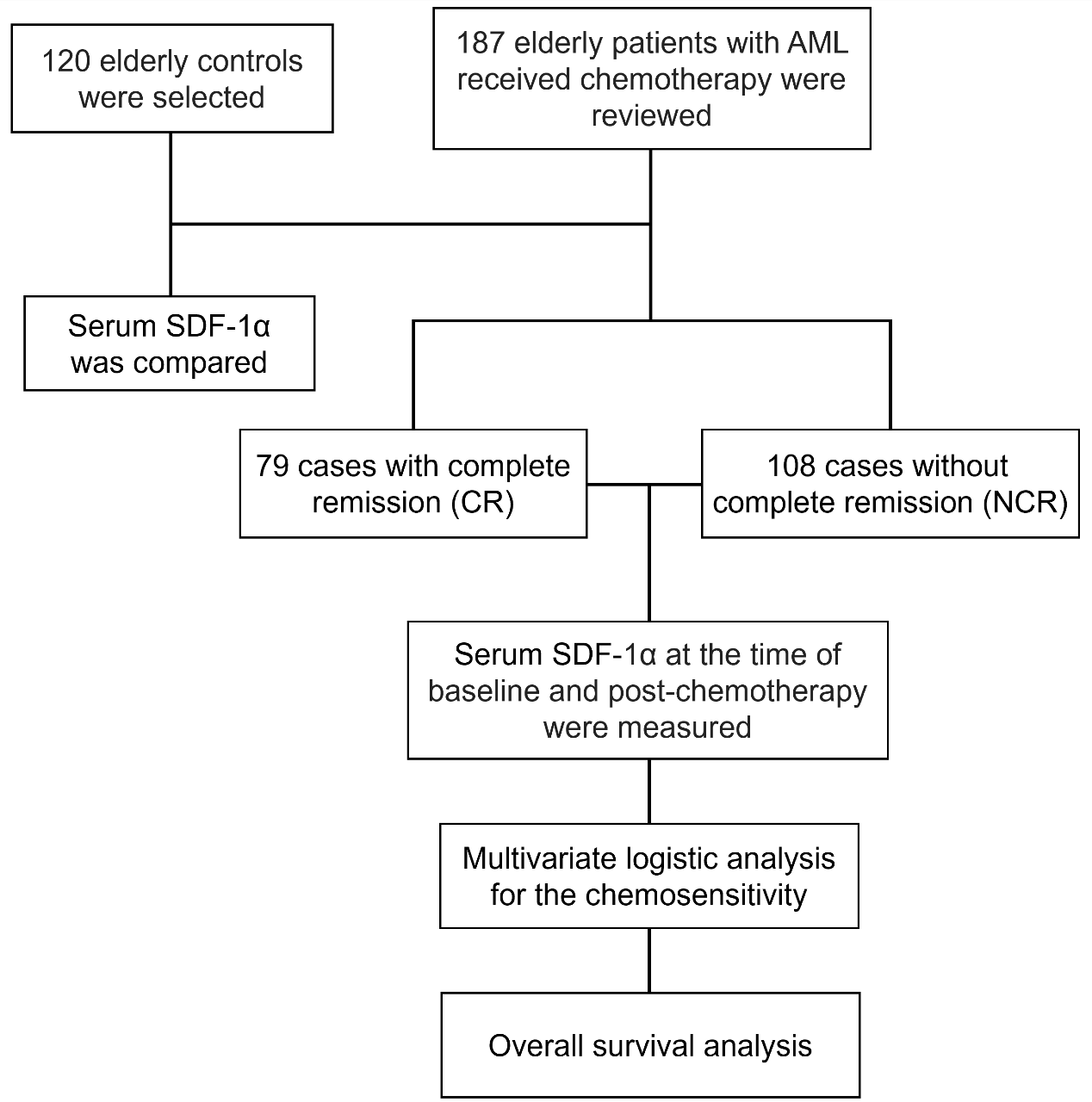


Figure S1. Flow-chart of the study.

Supplement: Supplementary file 1 [file DataSheet1.docx]
